# Preimplementation Evaluation of a Self-Directed Care Program in a Veterans Health Administration Regional Network: Protocol for a Mixed Methods Study

**DOI:** 10.2196/57341

**Published:** 2024-06-14

**Authors:** Pranjal Tyagi, Erin D Bouldin, Wendy A Hathaway, Derek D'Arcy, Samer Zacharia Nasr, Orna Intrator, Stuti Dang

**Affiliations:** 1 South Florida Veteran Affairs Foundation for Research & Education Miami, FL United States; 2 Division of Epidemiology Department of Internal Medicine University of Utah Eccles School of Medicine Salt Lake City, UT United States; 3 Providence Veterans Affairs Medical Center Providence, RI United States; 4 Canandaigua VA Medical Center Department of Veterans Affairs Canandaigua, NY United States; 5 VISN 8 Network Office Department of Veterans Affairs St. Petersburg, FL United States; 6 Department of Public Health Sciences University of Rochester Rochester, NY United States; 7 Geriatrics and Extended Care Data Analysis Center Canandaigua VA Medical Center Canandaigua, NY United States; 8 Miami VA GRECC Miami VA Healthcare System Miami, FL United States

**Keywords:** long-term institutional care, self-directed care, veteran directed care, veteran, veterans, institutional care, long term care, mixed-methods, caregivers, caregiver, United States, nursing home, homecare, community-based, home-based, unmet, pre-implementation, barriers, barrier, facilitators, facilitator, quantitative data

## Abstract

**Background:**

The Veteran-Directed Care (VDC) program serves to assist veterans at risk of long-term institutional care to remain at home by providing funding to hire veteran-selected caregivers. VDC is operated through partnerships between Department of Veterans Affairs (VA) Medical Centers (VAMCs) and third-party Aging and Disability Network Agency providers.

**Objective:**

We aim to identify facilitators, barriers, and adaptations in VDC implementation across 7 VAMCs in 1 region: Veterans Integrated Service Network (VISN) 8, which covers Florida, South Georgia, Puerto Rico, and the US Virgin Islands. We also attempted to understand leadership and stakeholder perspectives on VDC programs’ reach and implementation and identify veterans served by VISN 8’s VDC programs and describe their home- and community-based service use. Finally, we want to compare veterans served by VDC programs in VISN 8 to the veterans served in VDC programs across the VA. This information is intended to be used to identify strategies and propose recommendations to guide VDC program expansion in VISN 8.

**Methods:**

The mixed methods study design encompasses electronically delivered surveys, semistructured interviews, and administrative data. It is guided by the Consolidated Framework for Implementation Research (CFIR version 2.0). Participants included the staff of VAMCs and partnering aging and disability network agencies across VISN 8, leadership at these VAMCs and VISN 8, veterans enrolled in VDC, and veterans who declined VDC enrollment and their caregivers. We interviewed selected VAMC site leaders in social work, Geriatrics and Extended Care, and the Caregiver Support Program. Each interviewee will be asked to complete a preinterview survey that includes information about their personal characteristics, experiences with the VDC program, and perceptions of program aspects according to the CFIR (version 2.0) framework. Participants will complete a semistructured interview that covers constructs relevant to the respondent and facilitators, barriers, and adaptations in VDC implementation at their site.

**Results:**

We will calculate descriptive statistics including means, SDs, and percentages for survey responses. Facilitators, barriers, number of patients enrolled, and staffing will also be presented. Interviews will be analyzed using rapid qualitative techniques guided by CFIR domains and constructs. Findings from VISN 8 will be collated to identify strategies for VDC expansion. We will use administrative data to describe veterans served by the programs in VISN 8.

**Conclusions:**

The VA has prioritized VDC rollout nationwide and this study will inform these expansion efforts. The findings from this study will provide information about the experiences of the staff, leadership, veterans, and caregivers in the VDC program and identify program facilitators and barriers. These results may be used to improve program delivery, facilitate growth within VISN 8, and inform new program establishment at other sites nationwide as the VDC program expands.

**International Registered Report Identifier (IRRID):**

DERR1-10.2196/57341

## Introduction

Due to exposures during their service, veterans are at increased risk for a variety of health conditions, such as mental disorders such as depression and posttraumatic stress disorder which are notably higher than in the civilian population [1-3]. Additionally, higher frailty is also found among veterans with more severe wartime exposures [1-3]. Moreover, an estimated 7% of veterans are living with Alzheimer disease and Alzheimer disease–related dementias. Many veterans who need assistance with multiple daily activities such as eating, bathing, and dressing, or have severe cognitive impairment, may need care in institutional settings such as nursing homes [4,5]. However, most older adults prefer to age in their own homes rather than in institutional settings [6]. Institutional care is also costly [7-9]. In fiscal year (FY) 2018, the Department of Veterans Affairs (VA) spent US $6.1 billion on institutional care, a 21% increase compared to 2014 [9]. By 2037, these costs are projected to double [9]. As the number of veterans at risk for long-term institutional care (LTIC) increases, the VA Geriatrics and Extended Care (GEC) has focused on expanding the home- and community-based services (HCBS) available to veterans [10].

The VA’s Veteran-Directed Care (VDC) is one such HCBS program, which was established as a partnership between the VA and the US Department of Health and Human Services Administration for Community Living in 2008 [11]. The goal of this collaboration was to help veterans with disabilities of all ages and their families receive needed services in their own homes and communities. The program operates as a self-directed program that empowers veterans at risk of LTIC to choose their own long-term care providers and services [12]. In the VDC program veterans and their caregivers have direct control over the goods and services they receive; they can hire their own workers, including family or friends, to provide homemakers, and home health aide services focused on delivering personal care services in the home. Patients who meet eligibility requirements, which include a clinical assessment of their needs to establish the level of care needed and approval of an appropriate budget, undergo referral and enrollment in VDC. These steps are collaboratively completed by the VA staff and VDC Providers such as Area Agency on Aging, Aging & Disability Resource Centers, Centers for Independent Living, and State Units on Aging. The VDC providers assist the veterans in fulfilling their employer responsibilities. Both VA and VDC providers subsequently review and approve all program expenditures and regularly evaluate the veteran’s health and well-being. The management of the budget is done by third-party Financial Management Services (FMS) staff, who receive a monthly fee for these administrative duties, which also include processing payroll and taxes.

Previous research has suggested that similar self-directed services are associated with fewer unmet long-term care needs, improved patient satisfaction, and lower risk of adverse outcomes, including injuries, compared to other HCBS [13]. VDC enrollees have similar hospitalization rates and costs compared to users of other VA-purchased HCBS, despite VDC enrollees being more medically complex. Compared to other HCBS enrollees, VDC enrollees were more likely to receive aid and attendance benefits, to have a spinal cord injury, and to have higher health care costs [12-17]. VDC enrollees were less likely to have a VA-paid nursing home admission compared to veterans using other personal care services paid for by VA [14]. In addition, there were fewer potentially avoidable acute care admissions and emergency department visits among rural veterans enrolled in VDC (but not among urban VDC enrollees) [14]. Moreover, there is qualitative evidence that the VDC program is acceptable, and satisfaction among enrolled veterans is high with participants expressing that it has given them purpose and meaning [18]. Given current workforce shortages in the health care sector and especially in rural areas, the ability to hire family members and neighbors as paid caregivers through self-directed services may be a particularly effective way to surmount access challenges for veterans [19].

VDC is currently available at 70 of the 171 Veteran Affairs Medical Centers (VAMCs) and served approximately 7232 veterans in FY 2023, a nearly 15% increase from FY 2022. As a solution to meet its priority of allowing veterans to age in place if that is their preference, the VA has committed to expanding VDC to all VA facilities by the end of FY 2024 [20]. However, there is limited understanding of factors that affect VDC expansion and program-level needs to increase enrollment.

The objective of this pre-expansion implementation evaluation is to understand the factors that affect VDC program implementation and growth in current sites in Veteran Integrated Service Network (VISN) 8, to inform implementation in new sites. This study focused on 7 VDC programs within a single VISN. In the VA Healthcare System, VISNs include multiple VAMCs and Community-Based Outpatient Clinics and represent an important unit for oversight and service delivery [21]. VISN 8 cares for veterans in 7 VAMCs across Florida, Puerto Rico, and South Georgia and serves about 10% (n=619,840) of veterans aged older than 65 years receiving VA care in the country.

The primary aim of this project is to evaluate the VDC program implementation in VISN 8 with the following objectives: (1) describe the variability in VDC program organization and delivery across VISN 8, (2) identify barriers and facilitators faced by existing VDC programs in VISN 8, (3) understand leadership and stakeholder perspectives on VDC programs’ reach and implementation, (4) compare VDC programs in VISN 8 to national in terms of veterans served and GEC service use, and (5) use the information from aims 1-4 to identify strategies and make recommendations to guide VDC program expansion.

In this paper, we describe this study’s objectives and methods of this pre-expansion implementation evaluation.

## Methods

### Project Site

The VA Sunshine Healthcare Network is the nation’s largest system of VA hospitals and clinics serving a population of more than 1.5 million veterans in a vast area of 64,153 square miles spread across 79 counties in Florida, South Georgia, Puerto Rico, and the US Virgin Islands [22]. VISN 8 serves a substantial proportion of older veterans; in FY 2020, veterans aged 65 years or older comprised a little over 50% of the entire veteran population served in Florida [23]. Every VAMC in VISN 8 has an operational VDC program. The 7 VAMCs of interest for this study are located in Bay Pines, Gainesville, Miami, Orlando, Tampa, and West Palm Beach (Florida), and San Juan (Puerto Rico). The catchment areas for these VAMCs also cover a portion of Southern Georgia (Gainesville VAMC) and the US Virgin Islands (San Juan VAMC).

### Conceptual Framework—Consolidated Framework for Implementation Research

We used the Consolidated Framework for Implementation Research (CFIR) to guide our evaluation plan, data collection, and analysis [24]. CFIR is a well-known determinate framework used throughout the VA and health services research to identify and describe variables influencing implementation. CFIR is an appropriate framework for providing a grounded understanding of the barriers and facilitators to the expansion and implementation of VDC programs across multiple contexts by various stakeholders. The updated CFIR 2.0 framework provides a comprehensive classification consisting of 48 constructs and 19 subconstructs over 5 domains: innovation, outer setting, inner setting, individual characteristics, and implementation process [25]. We identified potentially relevant CFIR constructs to assess the key determinants impacting the VDC program’s operations, as well as the dynamics of organizational structure, implementation support, and other relevant domains. We then used these identified constructs to develop quantitative and qualitative data collection instruments and to guide analysis. Not all CFIR domains were represented in all the instruments (Table 1). This is consistent with other work using CFIR. Our iterative review process included project team discussions, consultation with a VA VDC staff member, and feedback from operational partners and another VA research team with expertise in VDC.

**Table 1:** CFIR (version 2.0)a domains and constructs represented in data collection by the study population.

Construct			VA^a^ leadership		VA staff		VDC^b^ providers (AAA^c^ and ADNAs^d^)		A_1_, enrolled veterans		B_1_, enrolled veterans’ caregivers		C_1_, enrolled veterans’ employees		A_2_, unenrolled veterans		B_2_, unenrolled veterans’ caregivers	C_2_, unenrolled veterans’ employees
**Innovation**																		
	Relative advantage			✓		✓		✓		✓		✓		✓		✓		✓
	Evidence base			✓		✓												
	Adaptability	✓		✓		✓												
	Complexity			✓		✓		✓		✓		✓		✓		✓		✓
**Outer setting**																		
	Partnerships and connections			✓				✓		✓		✓		✓		✓		✓
	Policy and laws	✓																
	Local conditions	✓						✓		✓		✓		✓		✓		✓
**Inner setting**																		
	Access to knowledge and information			✓		✓		✓		✓		✓		✓		✓		✓
	Work infrastructure	✓		✓		✓												
	Relative priority			✓										✓		✓		✓
	Relational connections			✓		✓		✓		✓		✓		✓		✓		✓
	Available resources			✓		✓		✓		✓		✓		✓		✓		✓
	Structural characteristics	✓		✓		✓		✓		✓		✓						
	Mission alignment	✓				✓												
	Information technology infrastructure			✓		✓		✓		✓		✓						
**Individuals**																		
	High-level leaders			✓		✓												
	Implementation facilitators	✓		✓		✓		✓		✓		✓						
	Innovation recipients					✓		✓		✓		✓						
**Implementation process**																		
	Assessing needs			✓		✓		✓		✓		✓		✓		✓		✓
	Assessing context	✓						✓		✓		✓		✓		✓		✓
	Reflecting and evaluating	✓		✓		✓		✓		✓		✓						
	Adapting			✓														
	Tailoring strategies			✓		✓												
	Engaging			✓		✓		✓		✓		✓		✓		✓		✓
**Antecedent assessments**																		
	Implementation climate			✓		✓		✓		✓		✓		✓		✓		✓
	Appropriateness	✓																
**Implementation outcomes**																		
	Anticipated implementation outcomes	✓						✓		✓		✓						

^a^VA: Veterans Affairs.

^b^VDC: Veteran-Directed Care.

^c^AAA: Area Agency on Aging.

^d^ADNA: Aging and Disability Network Agency.

### Study Period

This 3-year evaluation will use concurrent mixed methods to collect CFIR-based data about VDC implementation in VISN 8 in FYs 2022-2024 [26].

### Data Collection Procedures

We have conceptualized this project occurring in 5 phases (Table 2), beginning with interviews of VA and Aging and Disability Network Agency (ADNA) staff (phase 1), followed by VISN and facility leadership (phase 2). In phase 3, existing administrative data will be used to describe the VDC participants in VISN 8 and nationally and compare results in VISN 8 to national VDC results. Phase 4 will involve interviews with veterans and caregivers who have been referred to or enrolled in VDC. In phase 5, we will integrate information from phases 1-4 to identify strategies and make recommendations to guide VDC program expansion. Both primary and secondary data will be used in this project. Primary data will be gathered through (1) VA and ADNA staff surveys, (2) VA and ADNA staff interviews, (3) VISN leadership interviews, (4) VAMC GEC leadership interviews, (5) veteran and caregiver surveys, and (6) veteran and caregiver interviews. See Multimedia Appendices 1-6 for all interview guides being used as primary data collection materials. We will use secondary data from VA administrative data sources about VDC, which appear in the Veterans’ Health Administration Corporate Data Warehouse, and the Geriatrics and Extended Care Data and Analysis Center (GECDAC) data files [27]. The GECDAC collects and analyzes population-based data about GEC programs and services, providing evidence-based information to facilitate continuous quality improvement [27]. We will collect data from participants from both the inner setting (VA program staff and leadership) and the outer setting (ADNA and FMS staff, and veterans and caregivers).

**Table 2:** Data collection methods, purpose, and results.

Phase	Participant group	Data collection methods	Purpose	Results or summary reports
1	VA^a^ and ADNA^b^ VDC^c^ staff	Surveys Semistructured interviews	Gauge knowledge base, staff experiences and perceptions, and program operation	Report on variability in VDC program organization and delivery
2	VISN 8^d^ and VAMC^e^ leadership	Semistructured interviews	Gain insight into leadership support, priorities, and funding	Factors impacting VDC programs’ reach and implementation, and organizational facilitators and barriers
3	GECDAC^f^ VDC data	Secondary administrative data from different VA sources	Data regarding veterans served and the use of HCBS^g^	Quantitative description and comparison of VISN 8 to national VDC programs on access and HCBS use
4	Veterans and caregivers	Semistructured interviews Surveys	Learn from the lived experiences of enrolled and unenrolled veterans’ and their caregivers. Detailed needs, social determinants, and service use data	Factors affecting VDC enrollment decision, and satisfaction with enrollment processes Health, function, quality of life, unmet needs, other HCBS program use, and socioeconomic status
5	Research team	Integration of findings from aims 1-4	Use the information from aims 1-4 to identify strategies to make recommendations to guide VDC program expansion	Final report on the project summarizing data from all stakeholders to inform VDC expansion

^a^VA: Veterans Affairs.

^b^ADNA: Aging and Disability Network Agency.

^c^VDC: Veteran-Directed Care.

^d^VISN: Veteran Integrated Service Network.

^e^VAMC: Veterans Affairs medical centers.

^f^GECDAC: Geriatrics and Extended Care Data and Analysis Center.

^g^HCBS: home- and community-based services.

### Quantitative Methods

#### Surveys

We have developed VDC and ADNA staff surveys and interview guides based on CFIR constructs to collect information on VDC program design, administration, and staffing. The survey and interview questions were informed by previous VDC work and are adapted from the Organizational Readiness to Change Assessment developed by Sperber et al [28] and Helfrich et al [29]. The survey and interview guides were reviewed by a VA VDC coordinator at 1 VISN 8 site and by national experts in VDC and other VA HCBS. We created distinct surveys for VA and ADNA staff that covered similar topics but addressed their unique roles and responsibilities (see Multimedia Appendices 1-6).

VA staff surveys include questions about VDC program staffing, enrollment criteria, program size, referral sources, program goals and tracking, the ADNA partners, and the use of an external financial management system. We collect information about the respondents’ professional and work experience, including how long they have worked with veterans, in VDC, and other HCBS programs. We ask respondents to rate a variety of VDC program aspects including referral and enrollment processes; workflow, communication, relationships, and payments between the VA and ADNA; VDC quality; and the overall program operation and delivery. The rating scale includes “excellent,” “very good,” “good,” “fair,” and “poor” response options. We ask what respondents would need at their site to be well-equipped for a hypothetical 25% increase in VDC enrollment, providing them with some options such as more staff; more streamlined referral and enrollment processes; more funding or quicker reimbursements, etc, and space for up to 3 additional items the respondent may enter. Response options for this question include “yes,” “no,” and “not sure.” We will field the final surveys via Qualtrics, and participants will be emailed an invitation message explaining the purpose of the project along with a link to the survey.

In the interview with veterans and caregivers, we will ask about the need for VDC service, factors affecting VDC enrollment decisions, and perceptions of the VDC program’s enrollment process and quality. We will also invite veterans and caregivers to complete a survey that asks about their health, quality of life, function, unmet needs, and other HCBS program use, using the Home Excellence Research and Outcomes Center to Advance, Redefine and Evaluate Non-Institutional Caregiving surveys fielded by the Elizabeth Dole Center of Excellence for Veteran and Caregiver Research [30].

#### Administrative Data

We will explore veteran demographics and health characteristics, along with the use of other VA HCBS designed to support veterans with disabilities or long-term health care needs [30,31]. Veteran demographics and health characteristics will be retrieved from the Geriatrics and Extended Care Data & Analysis Center Core Files (GCF) [32]. The GCF is a data set that includes information on all veterans who used the VA in an FY. The GCF combines information from many VA and non-VA data sources, capturing health care use, costs, risk factors, and outcomes for each veteran. A new GCF file is created each FY, and we will use the GCF FY file that matches the FY of a veteran’s VDC enrollment date when compiling demographic and health characteristic information. Variables will include veteran age, gender, marital status, race, ethnicity, VA enrollment priority group, rurality of veteran’s residence, diagnosed health conditions, Minnesota case-mix level, predicted LTIC risk score, Care Assessment Need score, Nosos score, and JEN Frailty Index score [33-35]. We will extract data on chronic conditions including but not limited to dementia, cancer, stroke, diabetes, chronic obstructive pulmonary disease, paraplegia, congestive heart failure, chronic kidney disease (CKD), and spinal cord injury. These diagnoses’ indicators will be identified by using hierarchical condition category (HCC) Version 24-Community variables applied to combined VA and Medicare data. A veteran will be considered to have dementia if HCC indicators HCC51 (dementia with complication) or HCC52 (dementia without complication) are flagged. Similarly, cancer will be indicated if HCC8 (metastatic cancer and acute leukemia), HCC9 (lung and other severe cancers), HCC10 (lymphoma and other cancers), HCC11 (colorectal, bladder, and other cancers), or HCC12 (breast, prostate, and other cancers and tumors) are flagged. Congestive heart failure is indicated if MCVA_V24_HCC85 (congestive heart failure) is flagged, and CKD is indicated if MCVA_V24_HCC136 (CKD, stage 5), MCVA_V24_HCC137 (CKD, severe, stage 4), or MCVA_V24_HCC138 (CKD moderate, stage 3) are flagged.

Health care use data will be retrieved using the GECDAC Residential History File (RHF). The RHF uses data from VA, Medicare, Medicaid, and nursing home resident assessments to provide a daily summary of an individual’s health service use and location of care [36]. Using the RHF, we will extract data from all inpatient visits, emergency department visits, inpatient rehabilitation, VA or non-VA nursing homes, or home health care use 180 days before and 180 days post the VDC enrollment date.

### Qualitative Methods

#### Interviews

We have developed interview guides based on the CFIR constructs discussed below and as shown in Table 1. The interview guides are tailored for each type of participant (ie, VA staff, ADNA staff, VAMC GEC leadership, VISN leadership, enrolled veterans, caregivers of enrolled veterans, unenrolled veterans, and caregivers of unenrolled veterans). Interviews will focus on site and program-specific contexts and the facilitators and barriers to VDC program implementation and administration. We will request verbal consent from all participants before the interviews.

VA and ADNA staff interviews will cover 6 CFIR domains [24]. Survey responses will be reviewed and incorporated into our interview templates to allow the interviewer to inquire about specific ratings or information from the interviewee’s responses. The questions will ask about roles and responsibilities, enrollment and referral procedures, expansion barriers and facilitators, adaptations or best practices, local leadership support, available and needed resources, and their personal anecdotes about the VDC program.

VISN 8 administration and leadership interviews will include 7 CFIR domains. The questions will ask about their roles in overseeing program operations, comparisons to other noninstitutional care services, experiences with program expansion and initiation, and the most impactful aspects of VDC when advocating for medical center support.

Veteran and caregiver interviews will include 7 CFIR domains. The questions will ask about their experiences with the recruitment and referral processes, factors they considered when choosing (or not choosing) VDC, how the program has helped or hindered receiving care, and how VDC delivery could be improved.

Procedures for all interviews are similar. Interviews will be semistructured and conducted by at least two qualitatively trained project staff, including 1 facilitator and 1 dedicated notetaker. In the event 2 project staff are unable to attend due to scheduling conflicts, 1 facilitator will conduct the interview and the notetaker will watch the recorded interview to develop notes. Interviews will last about an hour, with leadership interviews lasting about 30 minutes, and will be conducted via Teams (Microsoft Corp). Veteran and caregiver interviews may be conducted by telephone as needed based on available technology access. Interview participants will be asked for their permission to record and transcribe the conversation; transcriptions will be created using Microsoft Teams’ built-in transcription function and edited by project staff using the audio recording for reference. Detailed notes will be taken, reviewed for completeness against transcripts, and then finalized.

#### Participant Recruitment

We will identify participants by their relationship to each of the 7 VISN 8 VDC programs. Each VDC program has a designated VDC program coordinator who oversees their local program; we will therefore recruit 7 VDC coordinator participants. The project will be presented in a VISN 8 call to all the VDC coordinators by the VISN 8 GEC manager to stress the importance of the project. Following that, the 7 VA VDC coordinators will be invited to participate via email, with follow-up in a week, with up to 3 invitations. These VA VDC coordinators will be asked to provide contact information for the ADNA staff with whom they work, and we will invite these 7 ADNA staff to participate. ADNA representatives will be recruited via email consistent with the VA coordinator protocol. We will interview the GEC leads at all VAMCs, and VISN leads from GEC and the Caregiver Support Program.

We will ask VDC coordinators at each VAMC to contact veterans who are eligible for interviews and ask their permission to share their contact information with our team. The eligibility requirements for these patients include their having undergone referral processes of VDC but include both those who decided to enroll as well as those who did not enroll. We will invite them to participate and schedule interviews, with the intended aim of interviewing a dyad of a veteran and their caregiver that is enrolled and unenrolled from each of the 7 sites, for a total of 14 interviews. We will ask veterans for their caregiver’s contact information if they have one and invite them to participate.

### Ethical Considerations

This evaluation was determined to be a quality improvement project by the VA Miami Research and Development Service and received an exemption from a full institutional review board review. Therefore, formal informed consent is not required. However, participants will be made aware of the interview process, their rights to stop the interview at any time; how evaluators plan to use the data being collected; and of the measures and processes that will be followed to ensure confidentiality.

All data will be collected with the permission of the participant. Interview notes, transcripts, and matrix analysis will be stored in a secure folder behind the Veterans’ Health Administration firewall. The folder will only be accessible to approved team members.

### Data Analysis

#### Quantitative Survey Data

Both survey and administrative data will be analyzed by calculating frequencies for categorical variables or means, SDs, medians, and IQRs for continuous variables. Responses to open-ended questions will be synthesized into key summary points. We aim to characterize the veterans served by VDC at each site in VISN 8 and to identify any potential differences, recognizing that the underlying veteran populations across the state may vary by many of the demographic and health characteristics that will be evaluated.

We will compare responses between groups of interest using chi-square tests for categorical responses and *t* tests (1-tailed) for continuous responses and consider any *P* value less than .10 to indicate a statistical difference. Given the small number of responses and our focus on learning about and describing VDC-related needs and experiences, we will not rely heavily on formal statistical tests.

#### Quantitative GECDAC Data Analysis

We will summarize VDC “participants” health care service use, including HCBS, inpatient visits, emergency department visits, inpatient rehabilitation, VA or non-VA nursing home, or home health care, 180 days pre- and post-VDC enrollment date in periods of 30 days in the first 3 months and then the most distant quarter 91-180 days before or post VDC enrollment, and compare these to national VDC data.

### Qualitative Data Analysis

Qualitative data from semistructured preimplementation interviews will be analyzed using rapid qualitative techniques guided by CFIR domains and constructs [37-41]. Interview notes and Teams transcriptions eliminate the need for traditional transcription processes and specialized qualitative analysis software. A structured interview summary template will be created based on each of the interview guides. Interview notes will be divided among team members and summary points will be derived for each interview. These summary notes will be compared across at least two qualitative team members and will be refined and finalized. Summary points will then be entered in individual rows of a Excel (Microsoft Corp) spreadsheet. Qualitative analysts will review summary notes and identify key concepts, which will be added to the matrix as column headers. Summary note entries will then be coded at the intersection of the row and column. Team members will review codes for discrepancies and develop consensus by adding new codes or splitting summary notes. These codes will be used to develop themes within the CFIR constructs. These themes will then be reviewed and discussed with the full analytic team during weekly meetings and analysis memos will be drafted to document relevant findings.

## Results

The current status of this project is ongoing through the end of FY 2024 (September 2024), with recruitment of participants and data collection having begun in October 2022. Data collection and data analyses are both ongoing; as of April 2024, we have recruited 7 VDC coordinators, 15 ADNA representatives, 1 FMS representative, and 10 pairs of veterans and caregivers who have been referred to VDC in the past.

We will calculate descriptive statistics including means, SDs, and percentages for survey responses. Facilitators, barriers, number of patients enrolled, and staffing will also be presented. Interviews will be analyzed using rapid qualitative techniques guided by CFIR domains and constructs. Findings from VISN 8 will be collated to identify strategies for VDC expansion. We will use administrative data to describe veterans served by the programs in VISN 8.

## Discussion

### Principal Findings

The possibility of making VDC available to veterans nationwide depends on identifying barriers and facilitators to VDC implementation and expansion. Given that veterans prefer not to be in institutional settings, there is a palpable interest in expanding care options to include VDC for these patients. This evaluation will fill a critical gap in the literature related to VDC implementation in existing programs across the VA health care network.

Our proposed evaluation has several strengths. Our project uses a mixed methods approach with quantitative data using both surveys and administrative data, and qualitative data using interviews. Moreover, data will be gathered not only through the engagement of VDC staff and leadership at multiple organizational levels, both within VA and their partnering community agencies, but also directly from veterans and caregivers on how VDC, in its current iteration, addresses their needs. This study will strengthen our understanding of the barriers and facilitators impacting VDC-eligible veterans and examine the factors that influence veterans to choose VDC or elect to use other HCBS.

Potential weaknesses of this study include that these practices can be unique to the VA ecosystem. Moreover, it is only studying 1 VISN alone in the VA, a VISN that has a higher proportion of older veterans than other VA regions, thus potentially affecting the availability of services. Therefore, these results may not be generalizable to non-VA self-directed programs, nor to other VA regions. We also anticipate challenges associated with recruitment, especially for veterans who chose not to enroll in VDC.

This examination is particularly timely as President Biden required the VA to expand VDC to all VAMCs by the end of FY 2025 via Executive Order 14095—“Increasing Access to High-Quality Care and Supporting Caregivers” [42]. Our results will guide not only VDC programs wishing to expand their VDC patient roster but for those VAMCs newly implementing VDC services for the first time. Our long-term aim is to use this work to inform best practices, and policy decisions for VDC.

## Appendix

Multimedia Appendix 1 Veterans Integrated Service Network (VISN) 8 Veteran-Directed Care (VDC) coordinator interview guide.

Multimedia Appendix 2 Veterans Integrated Service Network (VISN) 8 Veteran-Directed Care (VDC) community providers interview guide.

Multimedia Appendix 3 Veterans Integrated Service Network (VISN) 8 Veteran-Directed Care (VDC) financial management services interview guide.

Multimedia Appendix 4 Veterans Integrated Service Network (VISN) 8 Veteran-Directed Care (VDC) leadership interview guide (financial year 2022 Q1-Q3).

Multimedia Appendix 5 Veterans Integrated Service Network (VISN) 8 Veteran-Directed Care (VDC) enrolled veterans and caregivers interview guide.

Multimedia Appendix 6 Veterans Integrated Service Network (VISN) 8 Veteran-Directed Care (VDC) nonenrolled veterans and caregivers interview guide.

## Abbreviations

ADNA: Aging and Disability Network Agency
CFIR: Consolidated Framework for Implementation Research
CKD: chronic kidney disease
FMS: Financial Management Service
FY: fiscal year
GCF: Geriatrics and Extended Care Data & Analysis Center Core Files
GEC: Geriatrics and Extended Care
GECDAC: Geriatrics and Extended Care Data and Analysis Center
HCBS: home- and community-based services
HCC: hierarchical condition category
LTIC: long-term institutional care
RHF: Residential History File
VA: Veterans Affairs
VAMC: Veteran Affairs Medical Center
VDC: Veteran-Directed Care
VISN: Veterans Integrated Service Network

## References

[1] Bhattarai JJ, Oehlert ME, Multon KD, Sumerall SW (2019). Dementia and cognitive impairment among U.S. veterans with a history of MDD or PTSD: a retrospective cohort study based on sex and race. J Aging Health.

[2] Zimmer Z, Korinek K, Young Y, Teerawichitchainan B, Toan T (2022). Early-life war exposure and later-life frailty among older adults in Vietnam: does war hasten aging?. J Gerontol B Psychol Sci Soc Sci.

[3] Krishnan LL, Petersen NJ, Snow AL, Cully JA, Schulz PE, Graham DP, Morgan RO, Braun U, Moffett ML, Yu H, Kunik ME (2005). Prevalence of dementia among Veterans Affairs medical care system users. Dement Geriatr Cogn Disord.

[4] Selim AJ, Berlowitz DR, Fincke G, Cong Z, Rogers W, Haffer SC, Ren XS, Lee A, Qian SX, Miller DR, Spiro A, Selim BJ, Kazis LE (2004). The health status of elderly veteran enrollees in the Veterans Health Administration. J Am Geriatr Soc.

[5] Sarrazin MV, Rosenthal GE, Turvey CL (2018). Empirical-based typology of health care utilization by Medicare eligible veterans. Health Serv Res.

[6] AARP (2021). Where We Live, Where We Age: Trends in Home and Community Preferences.

[7] Chidambaram P 10 things about long-term services and supports (LTSS). KFF.

[8] Guo J, Konetzka RT, Manning WG (2015). The causal effects of home care use on institutional long‐term care utilization and expenditures. Health Econ.

[9] Veterans' Use of Long-Term Care Is Increasing, and VA Faces Challenges in Meeting the Demand. GAO.

[10] Geriatrics and Extended Care Home. U.S. Department of Veterans Affairs.

[11] Opportunities Exist to Improve Management of Noninstitutional Care through the Veteran-Directed Care Program. Department of Veterans Affairs OIG.

[12] Yuan Y, Thomas KS, Frakt AB, Pizer SD, Garrido MM (2019). Users of veteran-directed care and other purchased care have similar hospital use and costs over time. Health Aff (Millwood).

[13] Mahoney KJ, Mahoney EK, Morano C, DeVellis A (2019). Unmet needs in self-directed HCBS programs. J Gerontol Soc Work.

[14] Yuan Y, Thomas KS, Van Houtven CH, Price ME, Pizer SD, Frakt AB, Garrido MM (2022). Fewer potentially avoidable health care events in rural veterans with self-directed care versus other personal care services. J Am Geriatr Soc.

[15] Savla J, Sapra M, Hagemann L, Luci K (2021). Home and community based service use among veterans with dementia living in rural virginia. Innovation Aging.

[16] Coburn AF, Ziller EC, Paluso N, Thayer D, Talbot JA (2019). Long-term services and supports use among older Medicare beneficiaries in rural and urban areas. Res Aging.

[17] Roberto K, Savla J, McCann B, Blieszner R, Knight A (2022). Dementia family caregiving in rural Appalachia: a sociocultural model of care decisions and service use. J Gerontol B Psychol Sci Soc Sci.

[18] Mahoney EK, Milliken A, Mahoney KJ, Edwards-Orr M, Willis DG (2019). "It's Changed Everything": voices of veterans in the veteran-directed home and community based services program. J Gerontol Soc Work.

[19] White House Executive order on increasing access to high-quality care and supporting caregivers. The White House.

[20] Veteran Directed Care Expansion and Federal Updates [Slide show; Presentation slides]. U.S. Department of Veterans Affairs.

[21] Veterans health administration. Veterans Affairs.

[22] VA Sunshine Healthcare Network. US Department of Veterans Affairs.

[23] National Center for Veterans Analysis and Statistics. Veteran Population.

[24] Damschroder LJ, Aron DC, Keith RE, Kirsh SR, Alexander JA, Lowery JC (2009). Fostering implementation of health services research findings into practice: a consolidated framework for advancing implementation science. Implement Sci.

[25] Damschroder L (2022). The updated Consolidated Framework for Implementation Research based on user feedback. Implement Sci.

[26] Tariq S, Woodman J (2013). Using mixed methods in health research. JRSM Short Rep.

[27] Geriatrics and Extended Care Data Analysis Center. U.S. Department of Veterans Affairs.

[28] Sperber NR, Miech EJ, Clary AS, Perry K, Edwards-Orr M, Rudolph JL, Van Houtven CH, Thomas KS (2022). Determinants of inter-organizational implementation success: a mixed-methods evaluation of veteran directed care. Healthcare.

[29] Helfrich CD, Li Y, Sharp ND, Sales AE (2009). Organizational readiness to change assessment (ORCA): development of an instrument based on the Promoting Action on Research in Health Services (PARIHS) framework. Implement Sci.

[30] Dang S, Garcia-Davis S, Noël PH, Hansen J, Brintz BJ, Munoz R, Valencia Rodrigo WM, Rupper R, Bouldin ED, Trivedi R, Penney LS, Pugh MJ, Kinosian B, Intrator O, Leykum LK, Elizabeth Dole Center of Excellence for VeteranCaregiver Research Team (2023). Measuring the unmet needs of American military veterans and their caregivers: survey protocol of the HERO CARE survey. J Am Geriatr Soc.

[31] Dang S, Bouldin E, Hathaway W, Penney L, Panellas I, Intrator O, Nasr S (2022). A system level pre-implementation evaluation to inform program expansion of veteran directed care.

[32] Dally S, Wesgate S, Phibbs C, Kinosian B, Intrator O (2022). Guidebook for Use of the GECDAC Core Files, Version 2.

[33] Ruiz JG, Priyadarshni S, Rahaman Z, Cabrera K, Dang S, Valencia WM, Mintzer MJ (2018). Validation of an automatically generated screening score for frailty: the care assessment need (CAN) score. BMC Geriatr.

[34] Wagner T, Stefos T, Moran E, Cashy J, Shen M, Gehlert E, Ash S, Almenoffa (2016). Risk Adjustment: Guide to the V21 and Nosos Risk Score Programs. Technical Report 30. HERC.

[35] Kinosian B, Wieland D, Gu X, Stallard E, Phibbs CS, Intrator O (2018). Validation of the JEN frailty index in the national long-term care survey community population: identifying functionally impaired older adults from claims data. BMC Health Serv Res.

[36] Intrator O, Makineni R Geriatrics and Extended Care Data Analysis Center Residential History File (GECDAC RHF). VIREC Webinars.

[37] Dang S, Bouldin E, Hathaway W, Tyagi P, Penney L, Intrator O, Nasr S (2023). Veteran directed care: a mixed methods study to inform self directed care program expansion for Veterans Affairs (VA). Abstract submitted for professional paper presentation at The Gerontological Society of America?s Annual Scientific Meeting, Tampa, FL. November 8-12.

[38] Vindrola-Padros C, Johnson GA (2020). Rapid techniques in qualitative research: a critical review of the literature. Qual Health Res.

[39] Beebe J (2014). Rapid Qualitative Inquiry: A Field Guide to Team-Based Assessment.

[40] Koenig CJ, Abraham T, Zamora KA, Hill C, Kelly PA, Uddo M, Hamilton M, Pyne JM, Seal KH (2016). Pre-implementation strategies to adapt and implement a veteran peer coaching intervention to improve mental health treatment engagement among rural veterans. J Rural Health.

[41] Hamilton A Rapid qualitative analysis: updates and developments VA HSR&D cyberseminar.

[42] Lewinski AA, Crowley MJ, Miller C, Bosworth HB, Jackson GL, Steinhauser K, White-Clark C, McCant F, Zullig LL (2021). Applied rapid qualitative analysis to develop a contextually appropriate intervention and increase the likelihood of uptake. Med Care.

