# Supplementary material for: Preimplementation Evaluation of a Self-Directed Care Program in a Veterans Health Administration Regional Network: Protocol for a Mixed Methods Study
Source: JMIR Res Protoc. 2024 Jun 14;13:e57341. doi: 10.2196/57341 (PMC11214023; doi:10.2196/57341)
Supplement: Multimedia Appendix 1 [file resprot_v13i1e57341_app1.docx]

**Multimedia Appendix 1.** VISN 8 VDC coordinator interview guide.

**VISN 8 VDC Expansion Project**

**Dr. Stuti Dang-PI**

**VISN 8 VDC Coordinator Initial Interview**

### Interviewer Notes:

The questions below are intended to serve as a guide for your interview. You can change the wording slightly to fit your natural pattern of speaking, but the numbered questions should be asked in very similar language to what is written below. Probes do not have to be covered and do not need to be read; they are suggested topics if needed to help the interviewee provide more detail.

Before beginning the interview, it is important to **have the pre-interview survey on hand**. Several questions incorporate the response from the pre-interview survey so you may want to edit or write in their response in those items. If the interviewee did not complete the pre-interview survey, you will need to ask them to provide the relevant information from the survey first.

#### **Introduction**

Thank you again for sharing your time with us to help us learn more about the Veteran Directed Care (VDC) program in VISN 8. As a reminder, the purpose of this project is to collect information to inform the VISN’s VDC expansion efforts.

This interview will follow up and go into more depth on the answers you provided in your survey about your program, role, and opinions about your local VDC program. You may skip any questions you do not want to answer or stop the interview at any time. The information you share will be combined with survey data so the team can better understand the VDC programs at each site across the VISN.

Your individual responses will also be combined with those from other sites, and we will make every effort to assure that no single person or program can be identified in any reports we create from these data.

Do you have any questions?

*[respond to any questions or concerns before moving on]*

To assure we accurately document your responses, we like to record our interviews. Are you okay with us recording this conversation?

*[If no, proceed with interview questions, documenting responses in written notes. If yes, start recording and gain permission for recording at the start of the interview]*

#### **Permissions for recording prompt:**

Read: It is [date] and I am conducting an interview with a VDC coordinator in VISN 8. Do you have any objections to me recording our conversation? Your response to this question will not affect your ability to participate in this project.

| **Questions** | **Responses** |
| --- | --- |
| 1. Can you walk me through a typical enrollment into your VDC program? How are Veterans typically referred to the program? (What do the handoffs look like?) **networks and communication**   **See pre-interview survey for ratings about referral sources* |  |
| 1. If needed: I see from your survey that you use [X] criteria for Veterans to enroll in VDC. Can you tell me more about how you decided on those criteria?   **See* *pre-interview survey for eligibility criteria.* |  |
| 1. What is your communication or education strategy for getting the word out about the program to Veterans? **engaging**   ***** *See pre-interview survey for ratings about communication and marketing.* |  |
| 1. What are the key changes you have made to the VDC program to make it work effectively in your setting? |  |
| 1. How does the VDC program compare to other non-institutional care programs? relative advantage |  |
| - 1. Probe: Similarities and differences between VDC and other programs? |  |
| - 1. Probe: Advantages/disadvantages? |  |
| 1. What has been a key benefit to Veterans in receiving VDC through your program? |  |
| 1. What has been a key challenge for Veterans receiving VDC through your program? |  |
| - 1. Probe: Can you think of any way(s) to address this challenge? |  |
| 1. Do you feel supported in delivering this program? Rewarded?  **implementation climate** |  |
| 1. Has someone (or a team) outside of the local VDC program been helping you with implementing the program? Please describe including their roles and activities. **external change agents / champion** |  |
| 1. What are your current local priorities? How does the attention directed toward those priorities impact VDC? **relative priority** |  |
| - 1. Probe: To what extent has VDC implementation or expansion taken a backseat to other high-priority initiatives going on now? |  |
| 1. Have you had sufficient resources to implement and administer the VDC program?  **available resources** |  |
| 1. How has leadership been involved with the VDC program? **leadership engagement** |  |
| 1. Based on your survey response, you would need [X resources] to expand your current program by 25%. Could you tell me more about your answer? *If multiple needs identified*: What would you need most? |  |
| - 1. What would you need to expand with the current community partner/ADNA? |  |
| - 1. Probe: Is there anything else that comes to mind now that we are talking about your needs to expand? |  |
| 1. If you could change one aspect of your VDC program, what might it be? |  |
| 1. Can you describe the working relationships that are key in your VDC program?  **networks and communication**   **See pre-interview survey for ratings about community partner relationship and types of community partners.*  Talk about relationships with: |  |
| - 1. Community partner/agency (including leadership since they have sign-off authority for Veteran Care Agreements and disenrollments) |  |
| - 1. Other VA programs (e.g., primary care, HBPC) |  |
| - 1. Veterans and caregivers enrolled in VDC |  |
| 1. How has the billing and payment process been going? Have there been any challenges with the billing process, e.g., community partner reimbursement?   * *See pre-interview survey for ratings about financial processes and needs.* |  |
| 1. Based on your survey response, you said you [do/do not] track goals at your site, like [see survey response]. | ---DO NOT FILL IN |
| - 1. *If tracking*: How have you been tracking goals? How are you using the data you collect? **reflecting & evaluating** |  |
| - 1. *Whether tracking or not*: What do you think should be tracked? **reflecting & evaluating** |  |
| **Closing** | ---DO NOT FILL IN |
| Those are all the questions that I have. Is there anything else that you want to say about the VDC program? |  |
| Could you recommend any other individuals who are involved with the Veteran-Directed Care program at your site that you think we should interview? |  |

Thank you very much for your time.

Note taking conventions:

Notes from speaker’s point of view: “I” not she

Notes template ready to copy and paste into Excel. No returns. No bullets. Wendy review the work around

Decide on the level of summary.
