# Supplementary material for: Preimplementation Evaluation of a Self-Directed Care Program in a Veterans Health Administration Regional Network: Protocol for a Mixed Methods Study
Source: JMIR Res Protoc. 2024 Jun 14;13:e57341. doi: 10.2196/57341 (PMC11214023; doi:10.2196/57341)
Supplement: Multimedia Appendix 3 [file resprot_v13i1e57341_app3.docx]

**Multimedia Appendix 3.** VISN 8 VDC financial management services interview guide.

**VISN 8 VDC Expansion Project**

**Dr. Stuti Dang-PI**

**VISN 8 VDC FMS Initial Interview**

### Interviewer Notes:

The questions below are intended to serve as a guide for your interview. You can change the wording slightly to fit your natural pattern of speaking, but the numbered questions should be asked in very similar language to what is written below. Probes do not have to be covered and do not need to be read; they are suggested topics if needed to help the interviewee provide more detail.

Before beginning the interview, it is important to **have the pre-interview survey on hand**. Several questions incorporate the response from the pre-interview survey so you may want to edit or write in their response in those items. If the interviewee did not complete the pre-interview survey, you will need to ask them to provide the relevant information from the survey first.

#### **Introduction**

Thank you again for sharing your time with us to help us learn more about the Veteran Directed Care (VDC) program in VISN 8. As a reminder, the purpose of this project is to collect information to inform the VISN’s VDC expansion efforts.

This interview will follow up and go into more depth on the answers you provided in your survey about your program, role, and opinions about your local VDC program. You may skip any questions you do not want to answer or stop the interview at any time. The information you share will be combined with survey data so the team can better understand the VDC programs at each site across the VISN.

Your individual responses will also be combined with those from other sites, and we will make every effort to assure that no single person or program can be identified in any reports we create from these data. **Since there is only one FMS provider for all of VISN8 it will likely be possible for people to know where you work. So, again, you do not have to answer anything you do not want to answer.**

Do you have any questions?

*[respond to any questions or concerns before moving on]*

To assure we accurately document your responses, we like to record our interviews. Are you okay with us recording this conversation?

*[If no, proceed with interview questions, documenting responses in written notes. If yes, start recording and gain permission for recording at the start of the interview]*

#### **Permissions for recording prompt:**

Read: It is [date] and I am conducting an interview with an ADNA VDC staff member in VISN 8. Do you have any objections to me recording our conversation? Your response to this question will not affect your ability to participate in this project.

| **Questions** | **Responses** |
| --- | --- |
| 1. Can you walk me through your agency’s process of working with ADNAS in the VDC program? (What do the handoffs look like? What information is shared between you, the ADNA, and the VA, if any? If serving multiple VAMCs, are there differences between them?) **networks and communication**   **See pre-interview survey for ratings about referral sources* |  |
|  |  |
| - - 1. Are there documents or guidance you provide to ADNAs, Veterans, their caregivers, or VDC Coordinators as they enroll? If so, could you tell me about where these came from (e.g., did you develop them or did you receive them from another agency)? |  |
| 1. How have current billing and payment process been going? Have there been any challenges with the billing process, e.g., VA reimbursement or issues with your ADNA(s)?   **See pre-interview survey for ratings about financial processes and needs.* |  |
| 1. Can you describe the working relationships that are key in your work in support of the VDC program?  **networks and communication**   **See pre-interview survey for ratings about VA relationship and types of community partners.* |  |
| - 1. Talk about relationship with…      1. ADNA staff      2. VA staff (if applicable)      3. Veterans and caregivers enrolled in VDC (if applicable) |  |
| 1. Does your agency have documented standard operating procedures for the VDC program? |  |
| 1. If yes: Could you tell me about where this document came from? e.g., Administration for Community Living (ACL), your agency, VA, ADNA, etc. |  |
| 1. Would you be willing to share your SOP with us? |  |
| 1. What processes have you developed to make the program work effectively in your setting? |  |
| - 1. Do you subcontract with anyone for any aspects of the VDC program? |  |
| 1. How do your VDC processes or services compare to other HCBS programs you support?? relative advantage   **If time allows, look at AAA website before interview to determine which HCBS they offer and manage.* |  |
| 1. *If needed:* HCBS programs include adult day programs, respite for caregivers, home-delivered meals, or other services your agency provides to help older adults and people with disabilities remain in the community. 2. *Note:* Medicaid offers money follows-the person programs similar to VDC in some states. If the FMS works with any of these could also ask for comparisons. |  |
| 1. What has been a key benefit to VDC programs and/or ADNA’s of yourFMS services? To Veterans receiving VDC through your partner ADNA(s)? |  |
| 1. What has been a key challenge for ADNAs receiving FMS services? For Veterans receiving VDC through your partner ADNA(s)? |  |
| 1. Are there any specific types of ADNAs or groups of Veterans or their employees – such as those with limited technology access or certain types of care needs or limitations – who you feel are not being adequately served through the tools you use or provide for the VDC program right now? |  |
| a. Probe: How have you addressed these challenges? |  |
| 1. What is your involvement, if any, when a Veteran is discharged or when an employee disenrolls from the program? |  |
| - 1. *If FMS has interaction with Veterans or employees:* What are the reasons Veterans to disenroll from VDC or caregivers stop being employees, in your experience? |  |
| 1. Do you feel supported in delivering this program? Rewarded?  **implementation climate** |  |
| 1. What are your current organizational priorities? How does the attention directed toward those priorities impact VDC? **relative priority** |  |
| 1. Have you had sufficient resources to implement and administer services to the VDC program?  **available resources** |  |
| 1. Are there any quality improvement efforts happening within your agency regarding services to the VDC program?    1. If so, What are the goals of that? Are you tracking any data? **reflecting & evaluating**   * Data tracking is listed on the survey |  |
| 1. How has leadership been involved with the services provided for the VDC program? This could include your direct supervisor, agency director, or other decision-makers in the organization. **leadership engagement** |  |
| 1. VA is interested in expanding the VDC program by 25%. What would your agency need to service that expansion? Could you tell me more about your answer? |  |
| 1. Probe: What would you need most? |  |
| ***Secondary Priorities – If time allows*** |  |
| 1. Has someone (or a team) outside of your agency, ADNA(s), or VA partners been helping you with implementing the program? Please describe including their roles and activities. **external change agents / champion** |  |
| 1. How much of Veteran’s budget is typically spent on direct personal care services versus equipment? What is the approval process like for equipment requests, and how are you involved in it? What guidance do you have for these requests? How has that process been working? |  |
| 1. Are you doing satisfaction surveys with Veterans and caregivers enrolled in VDC? With ADNAs you serve?   *Note: Can follow up via email about satisfaction surveys* |  |
| **Closing** | ---DO NOT FILL IN |
| Those are all the questions that I have. Is there anything else that you want to say about the VDC program? |  |
| Could you recommend any other individuals who are involved with the Veteran-Directed Care program at your site that you think we should interview? |  |

Thank you very much for your time.
