# Supplementary material for: Preimplementation Evaluation of a Self-Directed Care Program in a Veterans Health Administration Regional Network: Protocol for a Mixed Methods Study
Source: JMIR Res Protoc. 2024 Jun 14;13:e57341. doi: 10.2196/57341 (PMC11214023; doi:10.2196/57341)
Supplement: Multimedia Appendix 4 [file resprot_v13i1e57341_app4.docx]

# **Multimedia Appendix 4.** VISN 8 leadership interview guide (FY 2022 Q1-Q3).

# **GEC Leadership**

# Introduction

Thank you for sharing your time with us to help us learn more about the Veteran Directed Care (VDC) program in VISN 8. The purpose of this project is to collect information to inform the VISN’s VDC expansion efforts.

The questions below ask for some basic information about your role, then we ask for your opinions about the VDC programs in VISN 8. You may skip any questions you do not want to answer or stop at any time. The information you share will help our team better understand the VDC program. Do you have any questions before we begin?

# Interview Questions

Full name: **Wende Phillips**

Job title: **Social Work Supervisor for Geriatrics and Extended Care (Supervisor for Orlando’s VDC Coordinator, Chair for Non-Institutional Care and Practice, Chair for Development Committee)**

| **Questions** | **Responses** | **Summary** |
| --- | --- | --- |
| 1. Can you tell us a bit about your role? (clinical experience, scope of oversight, administration, etc.) |  |  |
| - 1. How do you see the VDC program fitting in GEC programs at your VAMC? |  |  |
| 1. What are your goals for GEC? |  |  |
| - 1. How does VDC support or align with GEC goals? |  |  |
| - 1. How are those goals currently being tracked and measured? VDC specific? GEC goals overall? |  |  |
| - 1. Do you have any recommendations for how goals could be tracked differently/better? |  |  |
| 1. What is the perceived value of the VDC program? (cost-effectiveness, quality assessment, efficiency in reaching the target demographic, benefits, employee satisfaction, etc.)? |  |  |
| - 1. How does this compare to other Non-Institutional Care (NIC) programs? |  |  |
| 1. In your experience, what are the barriers and challenges for enrollment across programs? (Prompt: Veterans, providers, any and all…) |  |  |
| - 1. What is the decision-making process for making a referral to one program over another? |  |  |
| 1. We have noted that there is a lot of variability in VDC enrollment and allotted FTEs across VAMCs within the VISN; why do you think this variability exists? (case mix, rurality of regions) |  |  |
| 1. In your opinion, what are the limiting factors in expanding VDC by 25% VISN 8 wide? (staffing, funding, eligibility policy, state rules, etc.?) |  |  |
| - 1. How do you see your role in your VAMC’s VDC expansion? |  |  |
| 1. Do you feel you have the support of facility (VAMC) leadership for VDC? |  |  |
| - 1. For VDC expansion? |  |  |
| - 1. What evidence is needed to change leadership opinion/support for VDC? |  |  |
| 1. How important is it that VDC be expanded at your site/VISN8? |  |  |
| 1. Is there anything else you want to tell us about GEC and VDC? |  |  |

*Thank you for your time in support of this VISN 8 VDC expansion initiative.*
