# Supplementary material for: Preimplementation Evaluation of a Self-Directed Care Program in a Veterans Health Administration Regional Network: Protocol for a Mixed Methods Study
Source: JMIR Res Protoc. 2024 Jun 14;13:e57341. doi: 10.2196/57341 (PMC11214023; doi:10.2196/57341)
Supplement: Multimedia Appendix 5 [file resprot_v13i1e57341_app5.docx]

# **Multimedia Appendix 5.** VISN 8 VDC enrolled veterans and caregivers interview guide.

# VISN 8 VDC Enrolled Veterans Interview Guide

| Date: |  |
| --- | --- |
| Interviewer: |  |
| Note Taker: |  |
| Veteran Participant #: |  |
| Caregiver Participant #: |  |
| Employee Participant #: |  |
| Audio File Name: |  |
| Notes: |  |

**Interviewer Notes**: The questions below are intended to serve as a guide for your interview. You can change the wording slightly to fit your natural pattern of speaking, but the numbered questions should be asked in very similar language to what is written below. Probes do not have to be covered and do not need to be read; they are suggested topics if needed to help the interviewee provide more detail.

# Introduction

Hello! My name is ______ and I’ll be asking the questions today. And ______ is here to take notes.

Thank you again for sharing your time with us to help us learn more about the Veteran Directed Care (VDC) program. Your input is important to us and will be used to improve the VDC program for Veterans like you.

We will be asking you questions related to your experience with the VDC program and why you decided to enroll. You can skip any questions you do not want to answer. You can stop the interview at any time. Your responses will be kept confidential. We may include things you say in reports, but we will never use your name. Do you have any questions?

With your approval, we would like to record our conversation so that we can accurately document your responses. Your response does not affect your ability to participate in this project. Do you give your permission to record?

***If yes, start recording.*** If no, proceed.

# Recording Permission

Today is [date] and we are conducting an interview with a Veteran who decided to enroll in the VDC program. Do I have your permission to record our conversation? Thank you. Ok! Let’s get started.

Read **if the Veteran and caregiver and/or employee are present together.** We will be conducting this interview in sections. This first set of questions asks the Veteran’s experience with the VDC program. Then we will have a few questions for the caregiver and/or employee. Do you have any questions before we begin?

# Veteran-Focused Questions

The first set of questions is for the Veteran, so please answer these questions from the Veteran point of view.

| **Question** | **Response Notes** | **Summary** |
| --- | --- | --- |
| 1. Please tell me about your overall experience with the VDC program. |  |  |
| - 1. How did you learn about the VDC program? |  |  |
| - 1. How would you describe your relationship and communication with your VDC contact? |  |  |
| 1. What made you decide to enroll in VDC? |  |  |
| - 1. How did your use or participation in other programs impact your decision to enroll? |  |  |
| 1. How was your family or caregiver involved in your decision to enroll in VDC? |  |  |
| 1. What are your recommendations for improving the VDC referral/enrollment process? |  |  |
| 1. What have you found to be some advantages of the VDC program? |  |  |
| - 1. What about disadvantages? |  |  |
| 1. Who do you employ through the VDC? |  |  |
| - 1. How did you find the employee(s)? |  |  |
| - 1. Were they already providing care for you? In what capacity? |  |  |
| 1. How did you find the employee hiring process? |  |  |
| - 1. What made it easy? |  |  |
| - 1. What made it difficult? |  |  |
| - 1. Do you have any recommendations for improving the employee hiring process? |  |  |
| 1. What about the ongoing administrative employee management process? |  |  |
| - 1. Can you tell me about how you submit timesheets? |  |  |
| 1. What are your recommendations for improving the employee management process? |  |  |
| 1. How would you say hiring someone through VDC has impacted your family? |  |  |
| - 1. Can you tell me what this person does to help you? |  |  |
| 1. Are you satisfied with your VDC budget? Can you tell us why or why not? |  |  |
| 1. What about the special purchase process, what is that like? |  |  |
| - 1. Have any of your requests been denied? If yes, could you tell us more about that? |  |  |
| 1. Is there anything you feel you need more help with? Please tell us about what you need. |  |  |
| - 1. What types of services would be most helpful for you? |  |  |
| - 1. What do you wish you had that you don’t right now? |  |  |
| 1. If there is an issue or you need something related to the VDC program, who do you contact? |  |  |
| - 1. How would you describe your relationship and communication with this person? |  |  |

# Caregiver Questions

Ok. Thank you for sharing your experiences. We are now going to focus on the caregiver perspective. Please answer these questions form you point of view as a family caregiver.

| **Question** | **Response Notes** | **Summary** |
| --- | --- | --- |
| 1. How would you say VDC has impacted your family? |  |  |
| 1. From your caregiver point of view, what are your recommendations for how to improve VDC? |  |  |
| 1. As a caregiver, please tell us about anything you feel you need. |  |  |
| - 1. What types of services would be most helpful for you? |  |  |
| - 1. What do you wish you had that you don’t right now? |  |  |
| 1. What about the Caregiver Support Program? Have you used this or are you familiar with it? |  |  |
| - 1. *If turned CSP down:* What made you choose VDC over CSP VDC? |  |  |

# Employee Questions

Ok. Thank you for sharing your experiences. We are now going to focus on the employee perspective. Please answer these questions form you point of view as a VDC employee.

| **Question** | **Response Notes** | **Summary** |
| --- | --- | --- |
| 1. Do you have any problems with the payment process? |  |  |
| 1. From the employee point of view, do you have any recommendations for how to improve VDC? |  |  |
| 1. Have you been a paid caregiver in any other programs? And if so, how does VDC compare to these programs? |  |  |

# For All Participants

Thank you. This last question is for all of you.

| **Question** | **Response Notes** | **Summary** |
| --- | --- | --- |
| 1. Is there anything else you would like to tell us about your decision to enroll in VDC? |  |  |
| - 1. Veteran |  |  |
| - 1. Caregiver |  |  |
| - 1. Employee |  |  |

# Closing

Thank you very much for your time. We would like to send a follow-up survey that would take 20-30 minutes to complete. Your participation will greatly help our work improving care for Veterans like you.

**If yes:**  Great! We can send this survey in the mail, send you a website link by email, or schedule a time to go through the survey with you and record your answers. What do you prefer?

Thank you.
